# Peritoneal Hydatidosis: A Case-Based Discussion and Management Strategies

**DOI:** 10.7759/cureus.94543

**Published:** 2025-10-14

**Authors:** Attka Maryam, Haider Hilal

**Affiliations:** 1 Radiology, Rashid Latif Medical College, Lahore, PAK; 2 Radiology, Shaukat Khanum Memorial Cancer Hospital and Research Centre, Lahore, PAK; 3 School of Medicine, St. George’s University, True Blue, GRD

**Keywords:** abdominal pain, cysts, echinococcus, hydatid disease, peritoneum

## Abstract

This report presents the case of a 58-year-old male labourer from Kasur, Pakistan, with abdominal pain for two years, abdominal distension for one month, and altered bowel habits for six months. He had no comorbidities or reported exposure to dogs or sheep. Clinical examination revealed abdominal tenderness and percussion dullness. Laboratory investigations revealed haemoglobin of 11 g/dL, total leukocyte count of 9.6 × 10³/µL, and elevated *Echinococcus* IgG antibodies. Ultrasound and CT scans showed multiple hepatic, splenic, and peritoneal hydatid cysts, with characteristic honeycomb and spoke-wheel appearances. A large calcified cyst was noted inferior to the liver. The patient was treated with Albendazole. After three cycles of treatment, peritoneal cysts regressed significantly, while hepatic cysts persisted. Follow-up imaging confirmed disease regression, and surgical intervention was not required. This case illustrates a rare occurrence of peritoneal hydatidosis in Pakistan, emphasising the need for early recognition, accurate radiological diagnosis, and appropriate medical management. In resource-limited settings, albendazole therapy can yield favourable outcomes, reducing the need for surgery.

## Introduction

Echinococcosis is a parasitic infection that presents a major public health concern in many countries, including Pakistan [1]. Human infection usually results from consuming food or water contaminated with *Echinococcus* eggs [2]. While the liver and lungs are the most common sites affected, other organs, e.g., the spleen and the intestinal area, may occasionally be involved [3]. Peritoneal hydatidosis, however, is rare, representing only 5-16% of hydatid cysts globally [4].

This report discusses the case of a 58-year-old male from Kasur, Pakistan, diagnosed with peritoneal hydatidosis through ultrasound and CT scans. The aim is to enhance understanding of this uncommon manifestation, underline the distinct radiological features, and explore the challenges in management.

## Case presentation

A 58-year-old male from Kasur, Pakistan, presented with a two-year history of abdominal pain, particularly in the lower abdomen. Additionally, he reported abdominal distension over the last month and altered bowel habits for the past six months. He was a labourer of a low socioeconomic class with no history of diabetes, hypertension, tuberculosis, ischemic heart disease, surgical interventions, or exposure to dogs or sheep. There was no significant family history or medical history.

On physical examination, the patient appeared healthy with a pulse of 80 beats/minute and blood pressure of 140/90 mmHg. Abdominal examination revealed tenderness and dullness of percussion, while the rest of the physical examination was unremarkable. Laboratory investigations showed a haemoglobin level of 11g/dL (normal range: 13.5-17.5 g/dL), a total leukocyte count of 9.6 × 10⁹/L (normal range: 4.0-11.0 × 10⁹/L), and a serum *Echinococcus* antibody IgG level of 27.59 U (negative value: 0-8 U).

**Figure 1:**
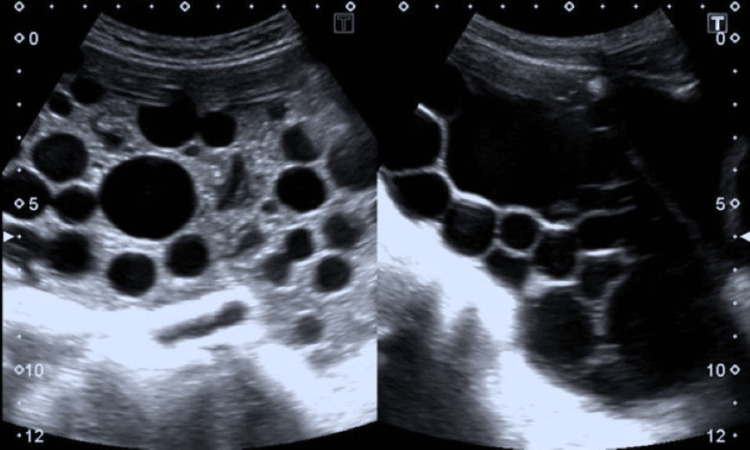
Peritoneal cavity with multiple cysts.

**Figure 2:**
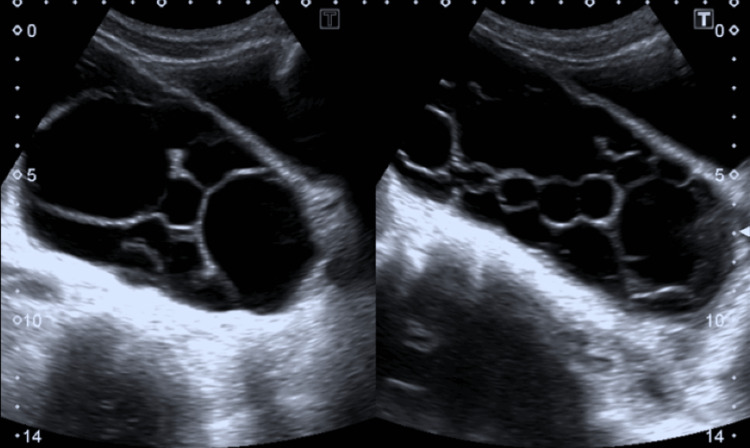
Pelvic ultrasound showing multiple cysts in the pelvic cavity.

Abdominal ultrasound revealed multiple large cysts in the liver and spleen. The peritoneal cavity exhibited numerous simple cysts, some containing daughter cysts (Figure 1). The liver had larger thin-walled avascular cysts, while the peritoneal cavity was filled with small avascular cysts. Thin-walled, irregular cystic lesions in the pelvis were present posterior to the urinary bladder (Figure 2).

**Figure 3:**
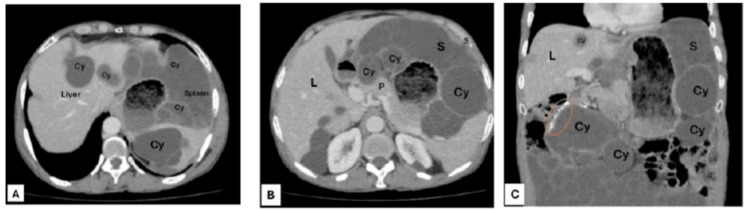
A. CT scan of the abdomen (axial section) showing hydated cysts (Cy) in the liver (L) and spleen (S). (B) Multiple multilocular fluid density lesions (Cy) anterior to the pancreas (P) and spleen (S), demonstrating a spoke wheel appearance characteristic of hydatid cysts (Cy). (C) A large, well-defined multivesicular cyst (Cy) inferior to the liver (L) showing thick peripheral wall calcification (orange circle). Other cysts (Cy) are also present inferior to the spleen (S).

The enhanced CT scan revealed compelling evidence of peritoneal hydatidosis in our patient. Within the liver and spleen, multiple well-defined cysts were observed (Figure 3A). Notably, the peritoneal cavity exhibited multivesicular hydatid cysts, forming a distinctive honeycomb pattern with thin, non-enhancing septa representing the walls of daughter cysts. These cysts in a group exerted pressure on the surrounding viscera without invasion. This resulted in the displacement of the small bowel to the left. Particularly striking were the multiple multilocular fluid density lesions, especially anterior to the pancreatic head and spleen, displaying a characteristic spoke wheel appearance indicative of hydatid cysts (Figure 3B). Additionally, numerous well-defined fluid density lesions were interspersed throughout the peritoneal cavity, providing further evidence of peritoneal hydatidosis. A prominent feature was the large, well-defined multivesicular cyst inferior to the liver, measuring 11.5 cm (anteroposteriorly) × 8.1 cm (laterally) × 6.9 cm (craniocaudally), exhibiting a thick peripheral wall calcification (Figure 3C). These detailed CT findings not only facilitated the diagnosis but also contributed crucial insights into the complex presentation and localisation of hydatid cysts in our patient.

**Figure 4:**
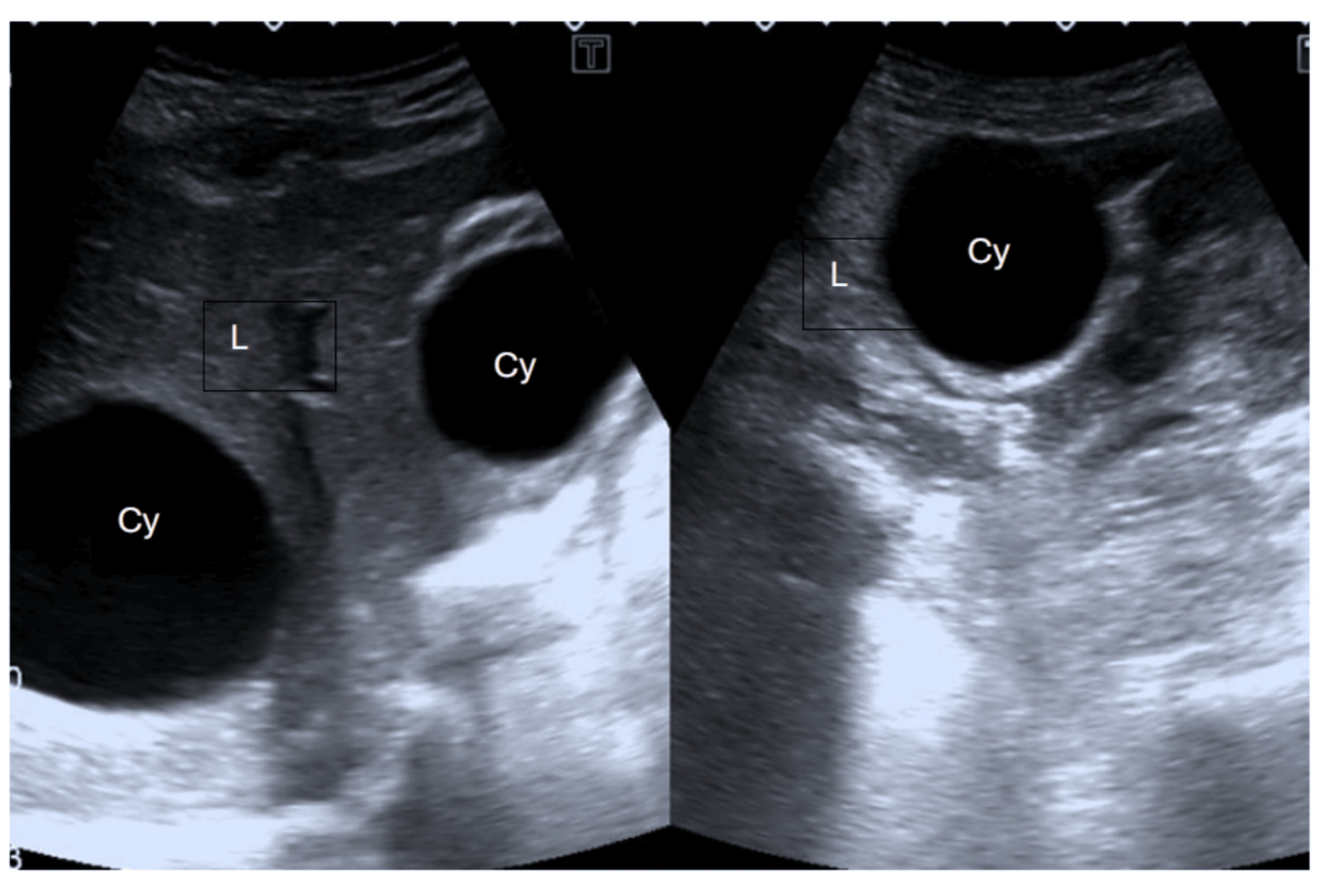
Ultrasound of the abdomen showing hydatid cysts (Cy) persisted in the liver (L) after treatment with albendazole.

**Figure 5:**
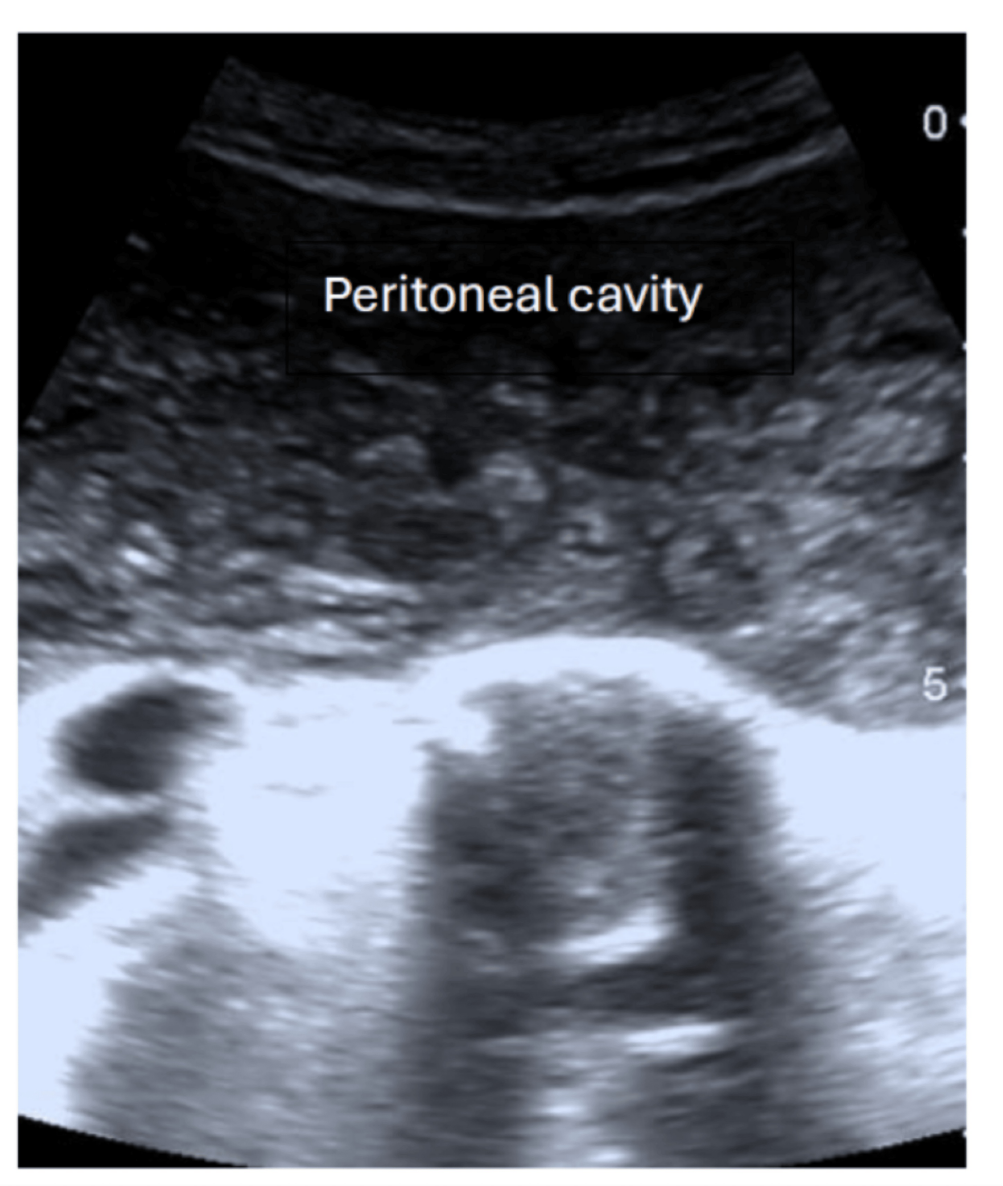
Ultrasound of the abdomen showing peritoneal cavity with no cysts after treatment.

**Figure 6:**
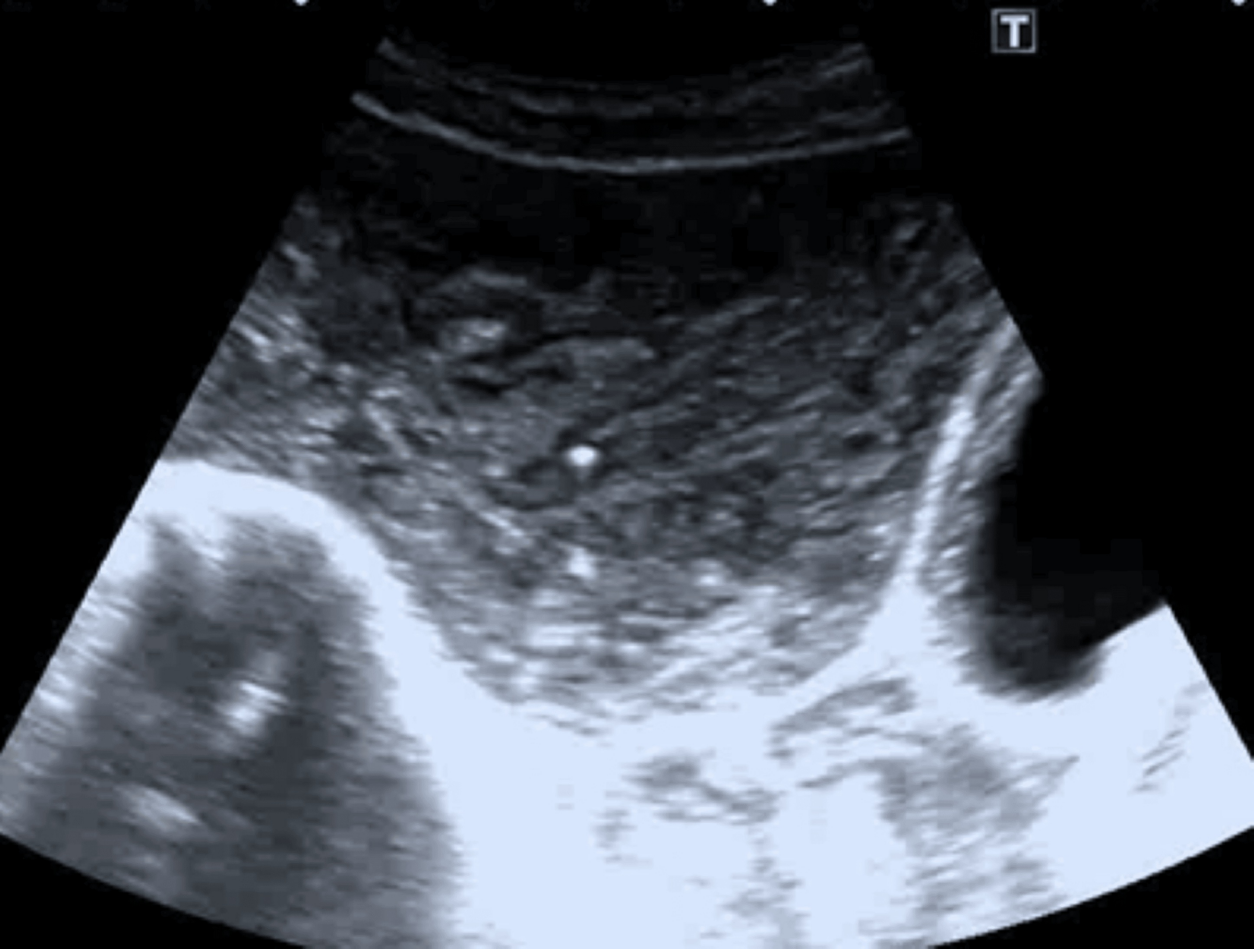
Ultrasound of the pelvis showing no cysts in the pelvic cavity.

The patient was treated with albendazole 200 mg, with three cycles and a 14-day gap in between. The patient showed improvement after three cycles of albendazole, with regression of multiple cysts in the peritoneal cavity. However, hepatic cysts persisted. Despite persistent hepatic cysts, the multiple cysts within the peritoneal cavity were no longer visible after treatment (Figures 4, 5). Ultrasound of the pelvis demonstrated the regression of previously seen large multivesicular cystic lesions (Figure 6). Surgical resection was deemed unnecessary, and the patient underwent follow-up.

## Discussion

Human echinococcosis is a zoonotic parasitic disease transmitted from canines to humans, most often through ingestion of parasite eggs in contaminated food or water [5]. Once ingested, the larvae disseminate via the bloodstream, primarily affecting the liver (70%) and lungs (20%), though less common sites such as the spleen, kidneys, and peritoneum have been documented [5].

In Pakistan, the disease is facilitated by close human-livestock-dog interaction, unhygienic farming practices, and limited healthcare infrastructure [6]. Our case highlights peritoneal hydatidosis, a rare manifestation. Similar presentations have been reported following spontaneous or iatrogenic rupture and in disseminated intra-abdominal disease [7-12]. The current case findings align with these reports, reinforcing the spectrum of atypical presentations documented in the literature.

Treatment strategies for cystic echinococcosis remain heterogeneous, with WHO guidelines recommending albendazole for small uncomplicated cysts, PAIR therapy for larger CE1/CE3a cysts, surgical or catheter-based approaches for CE2/CE3b cysts, and observation for inactive CE4/CE5 cysts [13]. Our management approach corresponds with these recommendations and mirrors outcomes described in previous studies, which demonstrate favourable results with combined medical and minimally invasive modalities [13,14].

Comparison with existing literature underscores that our case shares common features with other reported instances of peritoneal and disseminated hydatidosis [7-12,14], particularly in its clinical complexity and the need for individualised therapy. The successful management further supports evidence suggesting that integrating medical therapy with interventional or surgical techniques enhances outcomes and reduces recurrence risk [13,14]. This case contributes to the growing body of evidence on atypical hydatid disease, aligning with prior reports and reinforcing the importance of a tailored, guideline-driven approach. It also highlights the ongoing need for public health measures in endemic regions to interrupt transmission.

## Conclusions

This case highlights a rare instance of peritoneal hydatidosis in Pakistan, diagnosed in a 58-year-old male from Kasur. It emphasises the importance of awareness, timely diagnosis, and effective treatment strategies in resource-limited regions. CT imaging proved essential for diagnosis and follow-up, while albendazole therapy resulted in marked improvement. Given the limited data and healthcare barriers in Pakistan, echinococcosis remains underreported and underrecognized. Greater awareness, adherence to WHO guidelines, and improved surveillance are needed to address this neglected tropical disease effectively.
